# Small RNA-seq: The RNA 5’-end adapter ligation problem and how to circumvent it

**DOI:** 10.14440/jbm.2019.269

**Published:** 2019-02-20

**Authors:** Lodoe Lama, Jose Cobo, Diego Buenaventura, Kevin Ryan

**Affiliations:** 1 Department of Chemistry and Biochemistry, The City College of New York, New York, NY 10031, USA; 2 Biochemistry Ph.D. Program, The City University of New York Graduate Center, 365 Fifth Avenue, New York, NY 10016, USA; 3 Biology Ph.D. Program, The City University of New York Graduate Center, 365 Fifth Avenue, New York, NY 10016, USA; 4 Chemistry Ph.D. Program, The City University of New York Graduate Center, 365 Fifth Avenue, New York, NY 10016, USA

**Keywords:** small RNA-seq, coligo, TS2126, CircLigase, RNA ligase bias

## Abstract

The preparation of small RNA cDNA sequencing libraries depends on the unbiased ligation of adapters to the RNA ends. Small RNA with 5’ recessed ends are poor substrates for enzymatic adapter ligation, but this 5’ adapter ligation problem can go undetected if the library preparation steps are not monitored. Here we illustrate the severity of the 5’ RNA end ligation problem using several pre-miRNA-like hairpins that allow us to expand the definition of the problem to include 5’ ends close to a hairpin stem, whether recessed or in a short extension. The ribosome profiling method can avoid a difficult 5’ adapter ligation, but the enzyme typically used to circularize the cDNA has been reported to be biased, calling into question the benefit of this workaround. Using the TS2126 RNA ligase 1 (a.k.a. CircLigase) as the circularizing enzyme, we devised a bias test for the circularization of first strand cDNA. All possible dinucleotides were circle-ligated with similar efficiency. To re-linearize the first strand cDNA in the ribosome profiling approach, we introduce an improved method wherein a single ribonucleotide is placed between the sequencing primer binding sites in the reverse transcriptase primer, which later serves as the point of re-linearization by RNase A. We incorporate this step into the ribosomal profiling method and describe a complete improved library preparation method, Coligo-seq, for the sequencing of small RNA with secondary structure close to the 5’ end. This method accepts a variety of 5’ modified RNA, including 5’ monophosphorylated RNA, as demonstrated by the construction of a HeLa cell microRNA cDNA library.

## INTRODUCTION

**Figure 1.**
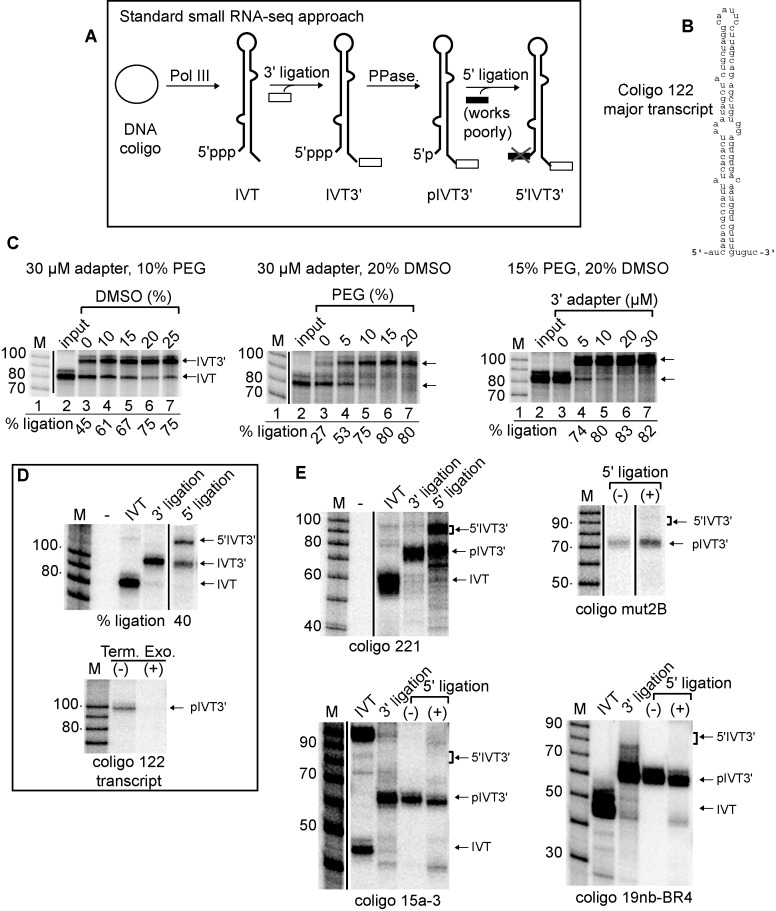
The 5’ sequencing adapter ligation problem with hairpin RNA. **A**. Schematic for the typical small RNA-seq approach using the addition of 3’ then 5’ adapters to, in our case, hairpin transcripts. IVT, Pol III *in vitro* transcript; Pol III, FLAG-tagged human RNA polymerase III; IVT3’, 3’ adapter-ligated transcripts; pIVT3’, 3’ adapter-ligated transcripts with 5’ phosphate; 5’IVT3’, fully adapter-ligated transcripts, 5’ppp, 5’ triphosphate; 5’p, 5’ monophosphate; PPPase, RNA 5’ polyphosphatase. **B**. Predicted secondary structure of the predominant coligo 122 transcript previously obtained using RACE-based method. **C**. Optimization of the 3’ adapter ligation reaction on coligo 122 transcripts under different DMSO (left gel), PEG (middle gel), or adapter (right gel) concentrations. Ligation percentage is defined as [ligated RNA/(ligated RNA + unligated RNA)] × 100. **D**. Upper gel: 3’ and 5’ adapter ligation evaluation for coligo 122. Lower gel: Verification of the 5’ monophosphate end of coligo 122’s pIVT3’ by Terminator exonuclease treatment (Term. Exo.). **E**. 5’ and 3’ adapter ligation evaluation for pre-miRNA-like hairpin transcripts related in predicted secondary structure to coligo 122 transcript, but unrelated in sequence (Sequences and predicted secondary structures have been verified and will be reported elsewhere). Brackets indicate the expected size of fully ligated products, 5’IVT3’. PEG, polyethylene glycol 8000; M, RNA Decade marker.

The standard approach to sequencing small RNA is to prepare a cDNA library by sequentially ligating 3’ and 5’ adapter DNA oligonucleotides of known sequence to the ends of the RNA [1-3]. Once flanking the region to be sequenced, the adapters provide primer annealing sites, first for the reverse transcription (RT) primer and later for the PCR and high throughput sequencing (HTS) primers (**Fig. 1A**) [2-4]. To avoid bias, *i.e.*, to ensure all RNA sequences are faithfully represented in the cDNA library, each sequence in the RNA mixture should undergo the library preparation steps equally well. Under enzymatic adapter ligation conditions, however, collections of diverse small RNA sequences may also include diverse RNA secondary structures, and some structures may be poor substrates for the adapter ligation reactions [5] or reverse transcriptase extension, for example. In very small RNA like the mature miRNA, or in fragmented mRNA, the size and/or sequence diversity of the ligation substrates reduce the likelihood of strong secondary structures, though even here bias can arise [6,7]. For other types of RNA, such as the tRNA and pre-miRNA, common secondary structures encompassing diverse sequences pose a systematic problem for the standard cDNA library preparation procedure [8-10]. The use of commercial small RNA cDNA library preparation kits has become widespread. In most kits, the success of the individual library preparation steps is not monitored, and thus bias due to structured RNA exclusion can escape notice. It is therefore important to characterize RNA structure-dependent problems that interfere with cDNA library preparation steps so that researchers can be alerted to the possibility of bias in advance, and so that improved methods to circumvent secondary structure-based problems can be developed.

We are investigating the use of circularized synthetic oligonucleotides, or coligos, as vectors for the ectopic expression of small RNA in human cells. As expression vectors, coligos are unique in that they contain only the template strand and lack a transcriptional promoter sequence, instead appearing to rely on structure-triggered RNA polymerase III transcription initiation and termination [11]. To date, we have designed coligos to encode transcripts resembling pre-miRNA with the aim that, when generated in cells, the transcripts might enter the natural miRNA maturation pathway and lead to mature miRNA mimics or siRNA. Pre-miRNA-encoding coligos produce hairpin transcripts with some 5’ and 3’ end heterogeneity, as judged by electrophoresis and limited complementary DNA (cDNA) sequencing analyses [11].

In order to understand the origins of coligo transcript end heterogeneity, we needed to characterize with precision the 5’ and 3’ ends of Pol III-coligo transcripts. When the standard small RNA cDNA library protocol failed for our pre-miRNA-like transcripts, we carefully monitored the individual library preparation steps and found that 5’ end adapter ligation using T4 RNA ligase 1 (T4 Rnl1) worked poorly for all coligo transcript substrates resembling pre-miRNA. Based on two previously sequenced examples, these coligo transcripts were all predicted to contain a single-stranded (ss) 5’end close to a hairpin double-stranded (ds) stem. We subsequently found two literature reports describing similar difficulties in the addition of the 5’ adapter to hairpin RNA. In one case the problem was suspected when a pre-miRNA isoform was detectable by northern blot but absent from a cDNA library [12]. Another report briefly described the difficulty in ligating a 5’ adapter to pre-miRNA isolated from cells carrying a catalytically inactive Dicer gene. Dicer inactivation was intentionally used to cause accumulation of the pre-miRNAs [10]. The problem in this case was characterized as resulting from the recessed 5’ end common to most pre-miRNA. In both of these examples, unusual experimental circumstances had led to the realization that there was a 5’ adapter ligation problem.

In the pre-miRNA report [10], although the 5’ adapter addition problem was not described in detail, it was circumvented by use of the ribosomal profiling method [13]. In ribosome profiling, the first cDNA strand, primed from the 3’ adapter, is circularized and then re-linearized at a different nucleotide in order to sandwich the cDNA region to be sequenced between two primer sites originating from the single (3’) adapter. In this report, we use several structurally similar RNA hairpin examples to illustrate the severity of the 5’ adapter ligation problem, and to expand its definition to include proximity of the 5’ end to a hairpin stem in the context of a 5’ ss extension, in addition to the 5’ recessed end context [10]. Poor 5’ ligation efficiency cannot be overcome by manipulating the reaction conditions, whereas we show that any difficulty in ligating the 3’ end adapter when close to the same hairpin could be overcome by varying the ligase reaction conditions. We adapt the circularization-relinearization approach of Ingolia *et al*. [13] and Liu *et al*. [10] to Pol III coligo transcripts, and improve the re-linearization step by replacing the original APE-1 relinearization reaction with simple RNase A hydrolysis at a single ribonucleotide placed in the RT primer. The key improvement in applying the ribosome profiling method [13] to pre-miRNA [10] was exchanging the biased 5’ adapter ligation step for the circularization step, but the circularization enzyme itself has been reported to introduce bias [14]. We investigate this by devising a unique ligation selection experiment and show that the circularization enzyme we use, TS2126 RNA ligase 1 (Rnl1), claimed recently to be the same as commercial CircLigase [15], has no significant bias in the positions surrounding the ligation site. Throughout the improved method that we describe, we show clear and careful monitoring of each library preparation step, ensuring that no step introduces strong or systematic bias.

## MATERIALS AND METHODS

### Coligo preparation

Linear coligo precursor oligonucleotides were purchased from Integrated DNA Technologies (IDT, Ultramer oligos) with 5’ phosphates. Sequences are shown in **Table 1**. Coligo, coligo precursor and coligo transcript secondary structures were predicted using the Mfold Web Server [16]. Coligo precursors discontinuous in regions predicted to be single-stranded (122 and Dcr3) were circularized using the TS2126 Rnl1 and isolated as previously described [17]. The preparation of coligo 19nb-BR4 is described elsewhere [18].

### Adapter and primer oligonucleotides

Custom sequencing adapters and primer oligonucleotides (**Table 1**) were ordered from IDT and purified using denaturing polyacrylamide gel electrophoresis (DPAGE). Adapters and primer sequences for library preparation and sequencing on Illumina MiSeq were based on the NEBnext small RNA library preparation kit (NEB, E7330S) with the following modifications: (1) no phosphorothioates were included ; and (2) an additional dC was placed at the 5’ end of Adapter3. During synthesis of LBprimer, the hand-mixing option was chosen. The 5’ radiolabeled LBprimer and reverse transcriptase primers (RTprimer1 and RTprimer2) used as tracers were prepared by sequential dephosphorylation with calf intestinal alkaline phosphatase (CIAP, Promega) and re-5’-phosphorylation in the presence of [γ-^32^P]-ATP by T4 polynucleotide kinase (NEB) according to the manufacturer’s protocol.

### *In vitro* transcription

FLAG-RNA Polymerase III (Pol III) was immunoprecipitated from HEK293/POLR3F stable cell line [19] lysates, and *in vitro* transcription reactions including a trace of [α-^32^P]-UTP were carried out on a 20 μl scale as previously described [18]. Transcription reactions (but not label) were scaled up 10-fold to produce the input for sequencing procedures.

### Optimized 3’ adapter ligation reaction

Coligo transcripts have a 5’ triphosphate, which blocks ligation side reactions. Therefore, the RNA transcripts made *in vitro* (10-fold scale-up) from a coligo template by immunoprecipitated FLAG-RNAP III were ligated to the 3’ adapter in a 20 μl ligation reaction that included the following components: 1 mM ATP, T4 RNA ligase buffer (NEB), 1 μl of T4 Rnl1 (NEB M0204S), 15% PEG 8000 (NEB), 20% DMSO, and 30 μM Adapter1 (**Table 1**). Ligation reactions were incubated for 2 h at 37°C. Nucleic acids were phenol/chloroform/isoamyl alcohol (PCI) extracted, ethanol precipitated and resuspended in RNase-free water for subsequent use. Note that to avoid undesirable side-reactions when using this method on 5’-monophosphorylated RNA, like miRNA and pre-miRNA, the 3’ adapter should first be 5’-pre-adenylylated, then ligated using the truncated T4 Rnl2(1-249) K227Q mutant without ATP [7].

### Library preparation procedure for coligo 122 transcripts followed by Sanger sequencing

A 10-fold scale up of the standard *in vitro* transcription (IVT) reaction using immunoprecipitated Pol III and coligo 122 as the template was carried out as previously described [18]. The reaction products were extracted with 1 ml Trizol reagent (Invitrogen) and included 10 µg glycogen. The precipitated transcripts were treated with RNase-free DNase I (NEB, M0303S, 4U) in 20 μl and incubated at 37°C for 30 min (to remove coligo template), PCI extracted and ethanol precipitated. The optimized 3’ adapter ligation reaction was carried out as described above, then PCI extracted and ethanol precipitated. The crude products were reverse transcribed using Superscript III (Invitrogen) according to the manufacturer’s protocol with following changes: 3 μM primer (RTprimer1) and incubation at 55°C for 1 h. A trace of 5’ ^32^P-labeled RTprimer1 was included to aid in cDNA visualization. At the end of the RT incubation, the reaction was treated with 5 units of RNase H (NEB, M0297S) at 37°C for 1 h, PCI extracted, ethanol precipitated and resolved on 10% DPAGE. The cDNA was located using Kodak Biomax XAR film exposure (Carestream, 1651454), excised, PCI extracted and precipitated. The gel-purified cDNA was circularized in 20 μl final reaction volume containing 50 μM ATP, 2.5 mM MnCl_2_, 50 mM MOPS (pH 7.5), 10 mM KCl, 5 mM MgCl_2_, 1 mM DTT, 3 μM TS2126 Rnl1 [17] at 60°C for 1.5 h [2]. Commercial CircLigase is reported to be the same enzyme [15] and may be used instead. The circularization products were PCI extracted, ethanol precipitated and re-linearized using RNase A (1 mg/ml final concentration in 10 μl TE buffer for 1 h at 37°C). The re-linearized cDNA was PCI extracted, ethanol precipitated, and then used as the template for PCR. The 50 μl PCR conditions were: 1× Taq polymerase buffer, 200 μM dNTPs, and 2.5 U Taq polymerase (Genscript, E00007), 24 cycles using 1 μM each Primer1F and Primer1R (**Table 1**) with denaturation at 94°C for 15 s, annealing at 58°C for 30 s, extension at 70°C for 30 s, and a final extension at 70°C for 5 min. The PCR products were resolved on a 2.5% agarose gel, and the bands visualized using ethidium bromide, excised and extracted using QuickClean II Gel Extraction Kit (Genscript, L00418). The extracted libraries were ligated into a linearized T-vector [20]. Five clones were picked and sequenced (Macrogen USA).

### Coligo-seq library preparation procedure for coligo Dcr3 with Illumina sequencing

The transcription, DNase I and 3’adapter ligation reactions were performed as described above but using Adapter3 as the 3’ adapter (**Table 1**). The products of the 3’ ligation reaction were purified by 10% DPAGE, located using a 2 h film exposure, excised, extracted and precipitated as described above. Gel purification at this point removes the need for RNase H after RT in this protocol. The gel-purified 3’ adapter ligated sample was reverse transcribed using Superscript III as described above using RTprimer2 (0.5 μM final concentration). It is important to use 55°C and to include a no-template control reaction to ensure RT products are template-dependent and not rare primer fold-back extension products. As before, a trace of 5’ ^32^P labeled primer was used for cDNA visualization. After RT, the sample was PCI extracted and ethanol precipitated. The products were resolved by 10% DPAGE, the cDNA was located by film exposure, excised, extracted and precipitated. Using the conditions listed above, the gel-purified end-labeled cDNA was circularized (TS21226 Rnl1), re-linearized (RNase A) and PCR amplified using Primer2F and Primer2R1 (**Table 1**) with the following modifications: 20 PCR cycles, denaturation at 94°C for 15 s, annealing at 62°C for 30 s, extension at 70°C for 30 s, and final extension at 70°C for 5 min. These primers introduced the Illumina flow cell P5 and P7 sequences. The PCR products were resolved on a 2.5% agarose gel, and the bands were visualized under UV light/ethidium, excised, and extracted using a Qiagen QuickClean II Gel extraction Kit and resuspended in water at 20 ng/ml. The coligo Dcr3 library was sequenced (110 cycles) on an Illumina MiSeq instrument at the Personalized Genomic Medicine Laboratory, Department of Pathology and Cell Biology, Columbia University Medical Center, New York, NY. A total of 2107614 Dcr3 sequencing reads were obtained. In keeping with transcripts made from a single coligo “gene”, only 8.48% of these were unique, and all variations were due to heterogeneity in the positions of Pol III initiation and termination. The raw Coligo-seq sequencing data for coligo Dcr3 transcripts have been deposited to the NCBI database with Biosample accession number SAMN07414566.

### Dcr3 Coligo-seq library sequence analysis

Bioinformatics analyses were done using tools freely available on the www.usegalaxy.org website [21]. Illumina MiSeq reads from the Dcr3 Coligo-seq library were selected based on the Dcr3 monomer transcript range (60–71 nt), which represents 89.41% of the total reads for the library. The ten most abundant Dcr3 whole transcript reads with a Phred score of 20 or higher were aligned using Clustal Omega [22]. To reveal precisely Pol III initiation and termination sites, 5’ and 3’ end analyses were performed. For the 5’ end analysis, the size-selected reads were trimmed to select the first 20 bases for each read. For the 3’ end analysis, the size-selected reads were trimmed to remove the single dC base from 3’ end of each read that had been added during library preparation. The reverse complement of each read was taken followed by trimming to select the first 20 bases from each read. The trimmed reads from both ends were filtered to retain those having a phred quality score of 20 or higher for each base in the read, leaving 90.2% (for 5’ ends) and 86.2% (for 3’ ends) of good quality reads with respect to total size selected reads. The filtered 5’ and 3’ end reads were then considered as the reference total for computing read coverage and for comparative analysis of preferred 5’ initiation sites and 3’ termination sites, and shown as percentages. To compute the preferred initiation and termination sites, the filtered reads were collapsed to find identical sequences and their abundance. In the Dcr3 sequence data, we found that a small percentage of reads mapped to the coligo dA larger loop initiation site but contained a non-templated (*i.e.*, non T) base, indicated by an asterisk. The template nucleotide was mainly read correctly as an A (27.4%), but in some reads incorrectly as G (3.5%), C (3.1%) or T (1.1%). One possibility is that the reverse transcriptase (RT) added an untemplated nucleotide to the 3’ end of the cDNA but the enzyme we used, Superscript III RT, is known to have very low terminal transferase activity. Thus, the source of these rare deviations is not yet known.

### TS2126 Rnl1 (a.k.a. CircLigase) circularization nucleotide bias analysis

DNA circularization reactions were carried out in a 20 μl volume as described above but with multiple concentrations of the 5’ phosphorylated DNA circularization substrate (LBprimer, **Table 1**). A trace of the ^32^P labeled LBprimer was included for visualization. Ligation reactions were incubated for 1.5 h at 60°C, followed by PCI extraction and ethanol precipitation. Precipitated samples were resolved by 12% DPAGE and circularized DNA from the reactions containing 0.25 and 0.5 μM LBprimer was recovered from the gel. The circularized DNA was re-linearized by treatment with RNase A as described above. One-half of the sample was used as template for PCR amplification (Primer2F and Primer2R1, 12 cycles). The PCR products were electrophoresed through a 2.5% agarose gel, the product band was located and recovered from the gel using Genscript QuickClean II Gel Extraction Kit and then sequenced on Illumina MiSeq (110 cycles). Atypically, only 3% (70072 reads) of the reads had a phred score of 30 or higher, indicative of high quality, while 6% had a score of 20 or higher. The data presented in the main text is based on these 70072 reads. As described in the Supplementary information, the unusually low percentage of high quality reads was caused by the small size of the library fragment that was sequenced. A second biological repeat with a modified PCR strategy corrected this problem, and yielded 94% high quality reads. Those data confirmed the results (see Supplementary Information).

## RESULTS

### Nearby hairpin RNA structure reduces 5’ adapter ligation efficiency

Our initial approach to deep sequencing coligo transcripts was based on the standard two-ligation small RNA-seq protocol (**Fig. 1A**). To illustrate the problem, we use a previously studied RNA hairpin transcript related to human pre-miR-122 [11] (**Fig. 1B**). This transcript was made *in vitro* by human FLAG-tagged Pol III, and contains a 5’ triphosphate (5’ppp). Using this transcript, we first optimized addition of the 3’ adapter by T4 RNA ligase 1 (T4 Rnl1) by varying DMSO, polyethylene glycol (PEG) 8000 and the concentration of the adapter (**Fig. 1C**). The optimized ligation reaction conditions, 20% DMSO, 15% PEG 8000 and 30 μM 3’ adapter, worked well for all coligo transcripts we have tried since, as shown for example in **Figure 1D** (top panel) and **Figure 1E**. After converting the 5’ triphosphate to monophosphate in preparation for adding the 5’ adapter, we were unable to reliably add a 5’ adapter using T4 Rnl1 (**Fig. 1E**). We verified that the polyphosphatase treatment had successfully converted the 5’ppp to 5’p by treatment with a 5’p-dependent 5’→3’ exonuclease (**Fig. 1D**, bottom panel). Unlike the 3’ adapter ligation, adjusting ligation conditions did not significantly improve the 5’ adapter ligation. Varations in DMSO, PEG, ATP and adapter concentrations, or reaction time, did not improve 5’ adapter ligation (**Fig. S1**). Each of the coligos studied in **Figure 1** encode RNAs similar in predicted structure to 122 (**Fig. 1B**) but entirely different in sequence. Our inability to reliably add the 5’ adapter to the coligo hairpin transcripts (**Fig. 1E**) suggested a general problem in adding a 5’ adapter to RNA with secondary structure close to the 5’ end (Each of these transcripts has since been verified to have a hairpin structure; manuscript in preparation). The difficulty we encountered in 5’ adapter ligation has also been noticed by others [10,12] but appears not to be widely appreciated. These results convinced us that the standard small RNA-seq method could not be used to characterize the end heterogeneity of coligo transcripts made by Pol III, which was our goal.

### Coligo-seq cDNA library preparation method

**Figure 2.**
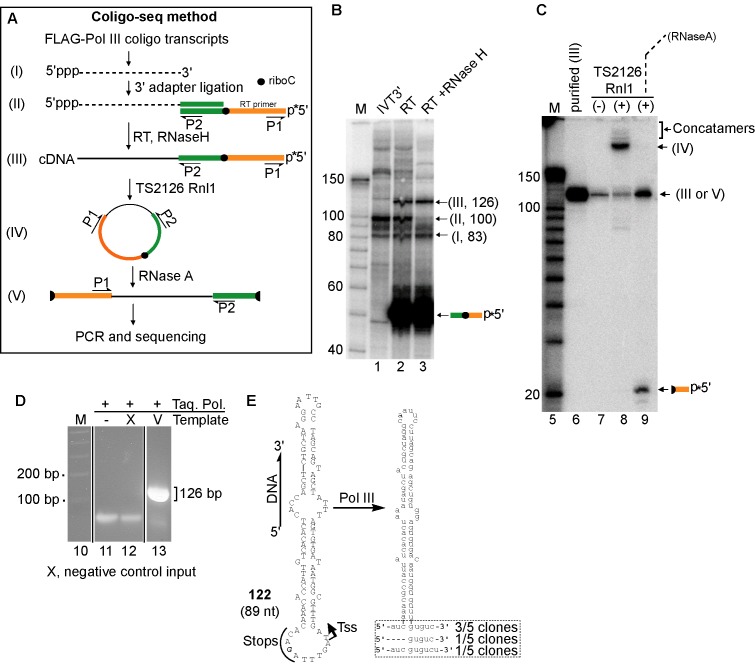
Coligo-seq proof of concept trial. **A**. Diagram depicting the Coligo-seq library preparation steps, followed by sequencing (in this experiment, Sanger sequencing). Black dot denotes single ribonucleotide and p* denotes 5’-^32^P label. Roman numerals are used to indicate different species produced during library preparation steps and their corresponding gel species in panels (B) and (C). P1 and P2, Primer1F and Primer1R. **B**. 3’ adapter ligation of coligo 122 transcripts followed by reverse transcription to make cDNA first strand using RTprimer1. 5’ radiolabeled cDNA (III) was excised for circularization. **C.** cDNA first strand circularization using TS2126 RNA ligase 1 (Rnl1) and re-linearization using RNase A. Re-linearized cDNA product (V) was used as the template for PCR amplification. Concatamers might include linear or circular tandem dimers. **D**. PCR amplification of re-linearized cDNA using Primer1F and Primer1R. The band indicated by bracket was cut, gel eluted, cloned, and sequenced. Templates used for PCR are: no template control (**-**), RTprimer1 negative control (X), where primer binding sites diverge, and re-linearized cDNAs (species V). **E**. Sanger sequencing analysis of the coligo 122 transcripts (5 clones total). Transcription start site (Tss, arrow) and transcript 3’ end site (Stops) were previously identified *via* 5’ and 3’ RACE sequencing are indicated for comparison.

To avoid this problem, we developed Coligo-seq, a single adapter ligation cDNA library preparation method summarized in **Figure 2A**. Following the addition of the 3’ sequencing adapter, Coligo-seq bypasses the troublesome 5’ adapter addition step and proceeds directly to RT using an RT primer containing the sequence information of both forward and reverse PCR primer binding sites (P1 and P2) in divergent orientations. This innovation was originally used in the ribosomal profiling method [13] where its need was probably less acute than it is for ligating adapters to structured 5’ RNA ends. It was later applied to pre-miRNA sequencing by Liu *et al*. [10]. Coligo-seq improves upon the existing method by placing in between P1 and P2 a single ribonucleotide residue (here, riboC). The reverse-transcribed cDNA is then circularized using the TS2126 Rnl1, a thermostable ligase we have used for a variety of tasks including coligo circularization [17] and adapter pre-adenylylation [23]. The circularized cDNA is efficiently re-linearized at the single riboC by RNase A treatment. Reopening the reverse transcript in between the two divergent primer sites creates a PCR template with the cDNA first strand placed between the now-convergent P1 and P2 primer sites. This PCR template can then be used for cloning or Illumina sequencing.

### Monitoring the Coligo-seq steps

In a preliminary experiment ending with Sanger sequencing, we subjected the *in vitro* transcription products made from coligo 122 by FLAG-Pol III to the Coligo-seq library preparation procedure while carefully monitoring each step (**Fig. 2**, Roman numerals designate each cDNA library species formed). The coligo transcripts were made with uniform [α-^32^P]-UTP incorporation. To visualize first strand cDNA synthesis, the RT primer, which includes the single riboC, was labeled at the 5’ end using T4 polynucleotide kinase and [γ-^32^P]-ATP. In **Figure 2B**, the reverse transcript (species III, lane 2) can be seen, and the RNase H-mediated removal of the transcript that served as the RT template is evident by comparing lanes 2 and 3 (species II). In this particular experiment, sub-optimal 3’ ligation conditions had been used, and some of the initial transcript was not ligated to the adapter (species I, lanes 1–3). The unligated RNA, though complementary to the cDNA produced, was not degraded by RNase H. This observation shows that RT produced a stable RNA:cDNA hybrid, and underscores the importance of optimizing the 3’ adapter ligation efficiency since sequence information in unligated transcripts will be lost at this step. The reverse transcript (species III), was extracted from the gel and circularized using TS2126 Rnl1 (**Fig. 2C**, lanes 6–8). The circularization reaction produced trace amounts of higher molecular weight concatamers. These products undergo digestion by RNase A to the same final PCR template (**Fig. 2**, species V). In our experience, the TS2126 Rnl1 ligase consistently circularizes with about 90% efficiency regardless of the DNA sequence (see later for a bias test for this step). The 10% of unligated species III (*i.e.*, the cDNA first strand) will not interfere with the PCR because RNase A will cut it between the P1 and P2 sites in the next step (**Fig. 2C**, lane 9, bottom of the gel). The circularized cDNA (species IV) was cleanly re-linearized by RNase A (lane 9) and used in a PCR reaction, which yielded a PCR product in the expected size range (**Fig. 2D**, lane 13, 126 base pairs, bp, expected). This DNA was recovered from the gel and cloned into a T/A cloning plasmid [20]. Five clones were examined by Sanger sequencing and three were found to be identical to the 122 transcript sequence found previously using modified 5’ and 3’ RACE-based methods [11] (**Fig. 2E**). This result demonstrated that the Coligo-seq approach could be used to sequence the cDNA of coligo transcripts without a 5’ adapter ligation step.

### A bias test for circularization by the TS2126 RNA ligase 1

**Figure 3.**
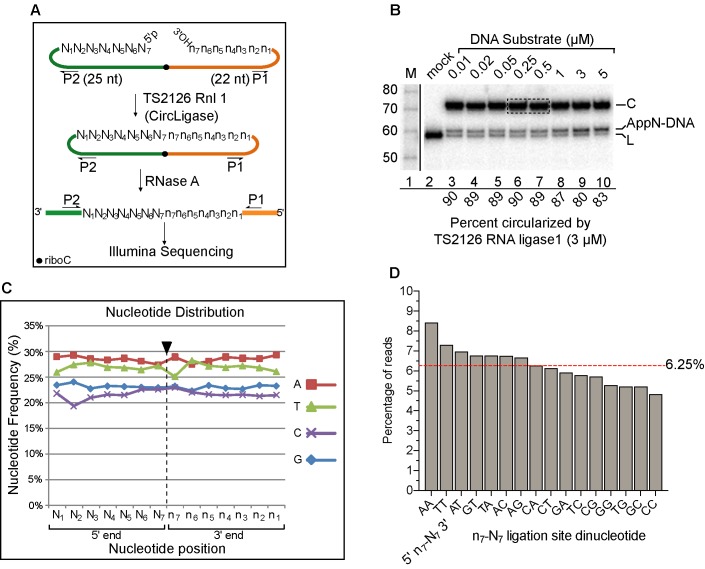
TS2126 Rnl1 circularization bias experiment. **A**. Library preparation scheme for high throughput sequencing using circular ligation substrate LBprimer. LBprimer consists of Illumina platform-compatible forward and reverse primer sequences separated by a single ribonucleotide (black dot) and flanked on both ends by seven randomized nucleotides. **B**. Circularization of LBprimer using 3 μM TS2126 Rnl1 with different substrate concentrations. L, linear LBprimer; C, circularized LBprimer; AppL, adenylylated linear LBprimer. Circularized products inside dashed box were recovered from the gel, pooled, re-linearized, PCR amplified (using Primer2F and Primer2R1) and sequenced on the Illumina MiSeq platform. Percentage circularized (%), listed below gel, is defined as [C/(C + L + AppL)] × 100. **C**. Nucleotide distribution found at each randomized position. **D**. Read frequency, by percentage, of all sixteen possible dinucleotides across the ligation junction. Dashed red line indicates the expected statistical percentage (100%/16 dinucleotides) that would result from unbiased ligation.

The construction of a cDNA library using Coligo-seq and other ribosome profiling-based methods replaces the unfavorable 5’ adapter ligation step with a cDNA first-strand circularization step. We have consistently observed intramolecular ligation yields of ~90% when circularizing ss DNA substrates of defined sequence [17,23], but during library preparation a circularization yield of < 100% could indicate bias. Furthermore, intermolecular ligation by commercial CircLigase, reported to be the same enzyme as TS2126 Rnl1(15) has been found to have sequence bias and erratic efficiency (14). To test whether the TS2126 Rnl1 ligase has circularization sequence bias, we constructed a TS2126 Rnl1 substrate library containing seven randomized nucleotides on each side of the ligation site (**Fig. 3A**). We also included the P1 and P2 PCR primer binding sites and, between them, a single riboC. Thus, this library uses a variation of the Coligo-seq strategy to identify sequences that have undergone successful ligation. Circularization by TS2126 Rnl1 at increasing substrate library concentration showed the typical ~90% circularization up to about 1 μM substrate (**Fig. 3B**). After isolation from the gel, the circularized library was re-linearized by RNase A and adapted for high throughput sequencing. The frequency of each nucleotide found at the fourteen positions is shown in **Figure 3C**. Throughout the randomized region, where each nucleotide should be represented at about 25% during DNA synthesis, there was a slight enrichment above 25% for A/T, and a slight reduction below 25% for G/C. However, throughout the randomized sequence the deviation from 25% at individual nucleotide positions was small, ranging from 23% to 26%. The 3’ terminal nucleotide showed almost equal frequency for G, C and T, and a slight preference for A. Importantly, all sixteen possible dinucleotide combinations were found to have been ligated and deviations from the predicted 6.25% (100%/16 dinucleotides) were small (**Fig. 3D**). A complete biological repeat confirming these results is described in the Supplemental Information, including **Figure S2**, **Figure S3**, **Figure S4** and **Table S1**. This result demonstrated that in the context of circularization, the TS2126 Rnl1 has no significant bias, and we conclude that for RNA with structured 5’ ends, exchanging the inefficient 5’ adapter ligation step of RNA-seq for the TS2126 Rnl1 circularization step of Coligo-seq offers a significant improvement. As noted above, the TS2126 Rnl1 is reported to be the same protein as the commercially available CircLigase [15], so these results should apply to that enzyme as well.

### Adapting Coligo-seq to high throughput DNA sequencing

**Figure 4.**
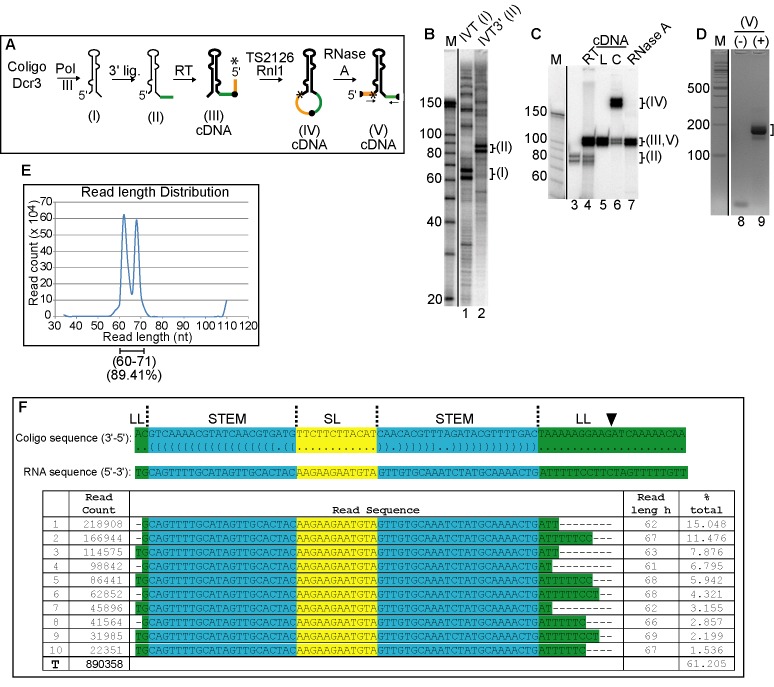
Monitoring Coligo-seq steps for Pol III transcription template Dcr3, and Illumina sequencing. **A**. Schematic representation of different species formed during library preparation. Asterisk, 5’-^32^P-end-label/ligation site; black dot, the single ribonucleotide in RT primer. **B**. Denaturing gel of *in vitro* transcription (IVT) and 3’ adapter ligation reaction products for coligo Dcr3 using Adapter3. **C**. Reverse transcription (RT) of gel purified 3’ adapter-ligated transcripts for cDNA synthesis using RTprimer2 followed by cDNA circularization and re-linearization with RNase A at the ribonucleotide site. L, linear cDNA first strand; C, circularization reaction product. **D**. PCR amplification of re-linearized cDNA template (V) using Primer2F and Primer2R2. Agarose gel. No-template control is indicated by (**-**). cDNA within the bracket was recovered for sequencing on Illumina MiSeq. **E**. Read length distribution of Dcr3 cDNA library after sequencing on Illumina MiSeq for 110 cycles followed by adapter trimming of each read. Horizontal bar below graph indicates the input RNA size range. Percentage of reads based on the total reads from the library. Compare peaks with transcript size in (B), lane 1. **F**. Whole-transcript read analysis for coligo Dcr3 cDNA library obtained after Illumina sequencing. The 10 most abundant reads are shown. Differently colored regions indicate different regions in predicted secondary structure of the coligo: larger loop (LL, green), smaller loop (SL, yellow), and stem including internal loops and bulges (cyan).

A second pre-miRNA-encoding coligo, Dcr3, based on the human pre-miRNA-19a cDNA template strand, was used to test adaptation of Coligo-seq to the Illumina HTS platform, again with careful monitoring at each step (**Fig. 4**). The Coligo-seq steps and species leading to the library PCR template are outlined in **Figure 4A**. Dcr3 was transcribed *in vitro* by Pol III, and the 3’ ends of the unpurified transcripts were ligated to the 3’ sequencing adapter under the optimized conditions (**Fig. 4B**). Note that all transcripts, including the higher molecular weight tandem repeat transcripts (formed by failure to terminate on the first or second circumtranscription), were shifted by efficient addition of the 3’ adapter. The 3’ ligation products corresponding to the two main circumtranscription products (~62 and ~67 nt, lane 1) were gel purified (**Fig. 4B**, lane 2, species II), extracted from the gel, reverse transcribed (**Fig. 4C**, lanes 3 and 4) and, after a second gel purification to remove excess RT primer (lane 5), circularized (lane 6), re-linearized by RNase A (lane 7) and amplified by PCR using primers containing sequences to adapt the cDNA library to the Illumina HTS platform (**Fig. 4D**). During sequencing, 2107614 reads were obtained. After removal of the primer sequences, 89.41% of the reads fell within the 60–71 nt size range of the original transcripts, and did so in two peaks (**Fig. 4E**) corresponding to the size of the excised transcript bands. This result demonstrated that about 90% of the Coligo-seq reads contained information from the size-selected Pol III transcripts.

**Figure 5.**
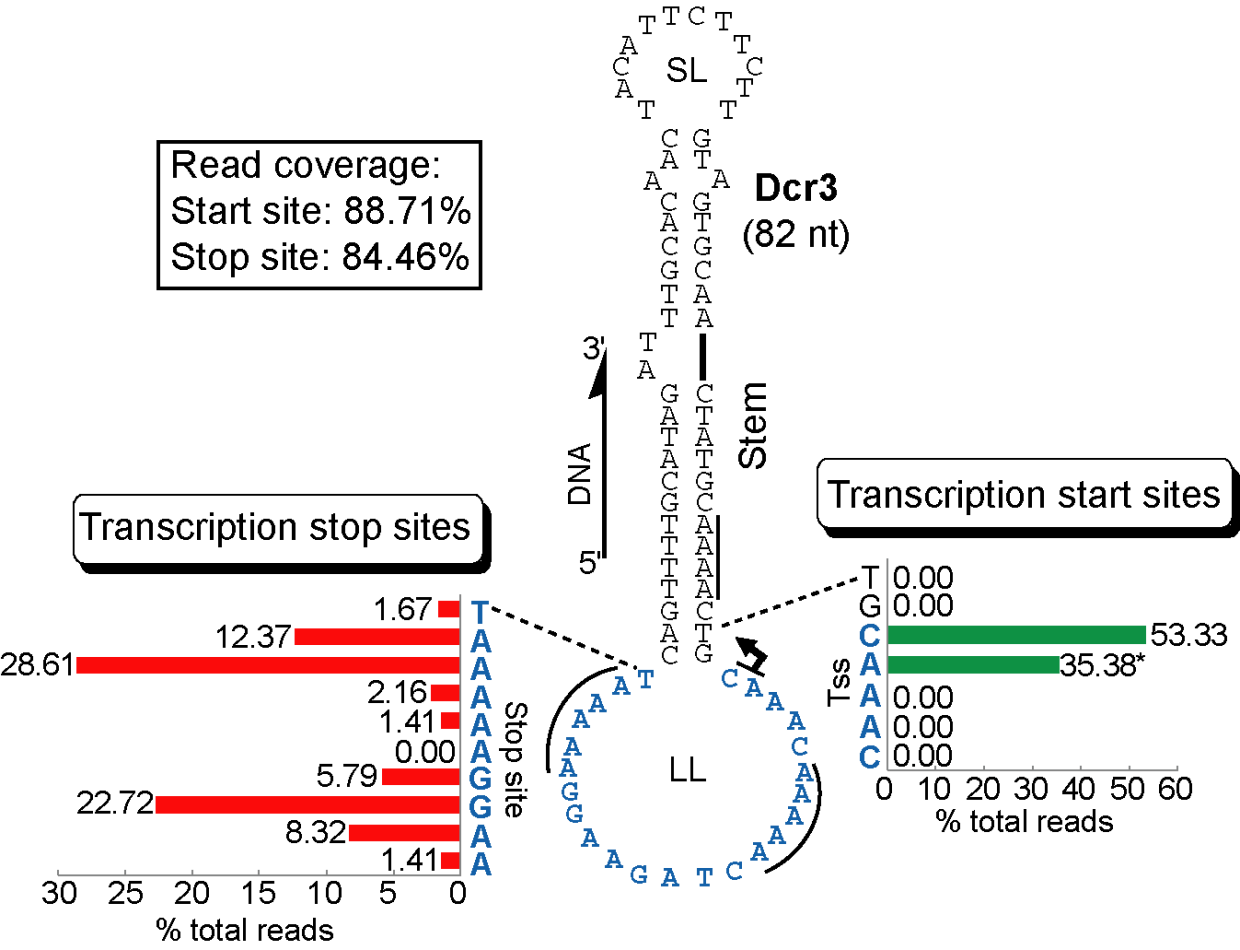
Coligo-seq results for Pol III template Dcr3 mapped to Dcr3’s predicted secondary structure. The transcription start and stop sites were compiled from the cDNA Illumina sequencing results and mapped to the predicted secondary structure. Reads representing less than 1% of the total were excluded, so the absence of a green or red bar indicates < 1% initiation or termination, respectively, at that template position. Solid lines over coligo sequence mark canonical Pol III termination signals. The asterisk (*) at the dA in the larger loop marks a start site position where a small percentage of the reads did not match the coligo dA (see Materials and Methods).

The ten most abundant Dcr3 reads, accounting for 61.2% of the total, are shown in **Figure 4F**. The percentage of reads for these ten transcripts would have been higher were it not for the occurrence of infrequent single nucleotide errors or mutations in the middle of the reads, far from the 5’ and 3’ ends we are aiming to analyze. These deviations from the Dcr3 sequence may arise through DNA synthesis errors, Pol III infidelity, or errors introduced in any of the Coligo-seq steps. **>Figure 4F** reveals the 5’ and 3’ end heterogeneity of the coligo transcripts, corresponding to Pol III initiation and termination events, respectively. The results show clearly that transcript heterogeneity comes mostly from the 3’ end. Because there was no evident correlation between the 5’ and the 3’ end sequences in these whole-transcript reads, we made the assumption that transcription starting and stopping occur independently of one another, and adopted the format of summarizing 5’ and 3’ end heterogeneity shown in **Figure 5**. Here, green bars and percentage numbers are used to denote the transcription initiation sites, and red bars with percentage numbers denote the transcription termination sites. The coligo is drawn with initiation site on the lower right, so that the colors need not actually be distinguished. Both ends are mapped to the predicted coligo secondary structure. Summarizing the sequencing data in this way confirmed our previous suspicion that the stem-larger loop junction is where most transcription begins and ends [11,18].

### Adapting Coligo-seq to 5’ monophosphorylated small RNA

**Figure 6.**
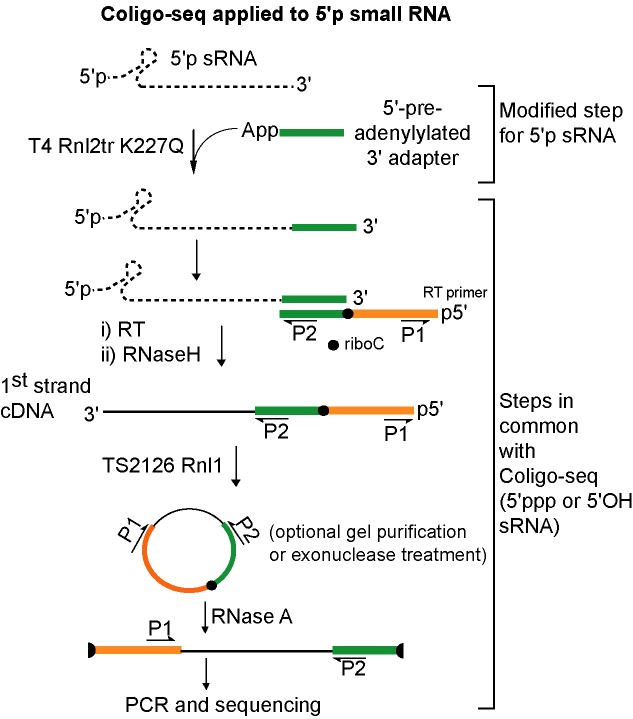
Coligo-seq variation for making a cDNA library from 5’ monophosphorylated small RNA.

Our method for small RNA cDNA library preparation can be easily adapted to small RNA from sources other than coligo transcription. If the RNA carries any modification besides a 5’ monophosphate, then no changes need be made to the procedure. However, during 3’ adapter ligation, a 5’ monophosphate on the source RNA can lead to transcript self-ligation side reactions such as multimerization or circularization [7]. To avoid these, the 3’ adapter should be pre-adenylylated [2,23] and the ligation carried out in the absence of ATP (**Fig. 6**). These changes, and the use of the modified T4 RNA ligase (T4 Rnl2tr K227Q, available from NEB) [7] suppress the side-reactions. All steps beyond this point are identical to Coligo-seq. To demonstrate this variation of Coligo-seq, we followed the **Figure 6** steps to make a cDNA library of mature miRNA isolated from HeLa cells. Mature miRNA carries a 5’ phosphate. We split a sample of total RNA and made a cDNA library from one half using Coligo-seq, and a cDNA library from the other half using the standard RNA-seq method [24]. The results are summarized in **Figure S5** and the miRNAs found in both cDNA libraries are tabulated in a searchable spreadsheet in **Table S2**. Out of the 186 most abundant miRNA cDNA reads found in the standard RNA-seq library, 184 were also found in the Coligo-seq library, and cases where a microRNA was found in one library but not the other were mainly limited to very low percentage reads, reflecting low abundance miRNA. These results validate the Coligo-seq variation shown in **Figure 6**. Thus, Coligo-seq can be used on small RNA regardless of the 5’ end modification, and provides a general method to circumvent the 5’ adapter ligation problem.

## DISCUSSION

The sequencing of small RNA is usually accomplished through construction and sequencing of the RNA’s cDNA library. While every step in the cDNA library preparation is a potential source of bias, it is the nucleic acid ligases needed to add the sequencing adapters that have generated the most concern [5,7,10,13,25]. In small RNA, where the chance for long stretches of base-pairing is possible but unlikely, sequence bias can become the problem [5,10,13]. However, when secondary structure common to a class of small RNA is important for its biogenesis or function, as in the case of the tRNA and pre-miRNA, the secondary structure common to the RNA may cause severe bias. The Pol III coligo templates we are studying code for RNA that resemble pre-miRNA hairpins. Liu *et al*. found that the 5’ adapter could not be ligated to cellular pre-miRNA hairpins using the standard RNA-seq protocol. They traced this ligation difficulty to the secondary structure and, in particular, the recessed 5’ ends common to pre-miRNA [10]. We found a similar difficulty when trying to ligate the 5’ adapter to coligo transcript hairpins, which have similar secondary structure but do not have a recessed 5’ end. Our observation that T4 Rnl1, the standard enzyme used for 5’ adapter ligation, failed to work on coligo hairpin transcripts has helped us to better define the 5’ adapter ligation problem: it is the proximity of the RNA 5’ end to a helical region that interferes with T4 Rnl1 ligation, whether the 5’ end is recessed or at the end of a short ss extension.

Like Liu *et al*. [10], we found that the 5’ adapter ligation problem prevented use of the standard small RNA-seq protocol to sequence our transcripts, and the only solution was to avoid 5’ ligation. The ribosome profiling method of circularizing the first strand cDNA, then reopening it at a different location, circumvents the 5’ ligation problem. In the original ribosome profiling method, cDNA circularization was followed by re-linearization at an RT primer abasic site using the apurinic endonuclease APE-1. Re-linearization prevents Taq polymerase from running around the cDNA circle multiple times during subsequent PCR, which could lead to heterogeneous PCR products. Some variations on the original Ingolia method have left the cDNA in circular form during PCR, rather than re-linearizing it [26], among other variations [27]. If left in circular form, the PCR reaction that generates the library used in the Illumina flow cell must be carefully monitored and stopped before high molecular weight PCR concatamers overtake the reaction [26]. In our experience, re-linearization produces a better PCR template for preparing the flow-cell sequencing template, and RNase A treatment is a rapid, efficient and inexpensive alternative to APE-1 and its modified DNA substrate.

RNA-seq methods that include a cDNA circularization step, like those of Ingolia *et al*. [13] and Liu *et al*. [10] have relied on the commercially available CircLigase enzyme, whereas we have used the TS2126 Rnl1. CircLigase was recently identified as TS2126 Rnl1 [15], though we cannot know if they have identical sequences. CircLigase has become widely used, but to our knowledge, only limited information is available on its bias [14,28]. We therefore designed an assay based on Coligo-seq to test for sequence bias surrounding the ligation site. Our results provide evidence that when working intramolecularly it has no significant sequence bias in the seven nucleotides on either side of the ligation site. Importantly, all sixteen possible dinucleotide combinations were found to have been ligated, and deviation from the predicted 6.25% (100%/16 dinucleotide combinations) was small. In contrast, when working intermolecularly, CircLigase showed strong bias against certain nucleotides at the ligation site [14]. The difference between those and our results appears to confirm the finding [29] that the TS2126 Rnl1 excels at circularizing ligation reactions, and is therefore well-suited to ribosome profiling-based RNA-seq strategies.

Though the Coligo-seq method we describe here is closely related to the earlier Ingolia *et al*. [13] and Liu *et al*. [10] library construction methods, the results we report here have important differences. First, we demonstrate how to optimize the three variables (DMSO, PEG and adapter concentration) important for efficient 3’ adapter ligation to RNA hairpins resembling pre-miRNA. Successful 3’ adapter ligation depends on the proper adjustment of these concentrations, but 5’ adapter ligation problems due to secondary structure were not overcome by similar adjustments. Second, we use several specific hairpin RNA examples to illustrate the difficulty of ligating the 5’ adapter, and which further define the 5’ adapter ligation problem by expanding it beyond 5’ recessed ends. Third, we show that as a workaround for problematic 5’ ligation, TS2126 Rnl1 circligation works with about 90% efficiency and shows no bias with regard to nucleotide identity at and around the ligation site. The data from that experiment should be of value to those using CircLigase in other applications. Coligo-seq also introduces a novel method for re-linearizing the cDNA by RNase A, and this strategy may be useful in other DNA selection experiments. Additionally, in our study of coligos as small RNA expression vectors, Coligo-seq now enables us to routinely sequence coligo transcripts so that we may define the rules for controlling Pol III initiation and termination on synthetic oligonucleotides.

RNA-seq protocols that profile mRNA typically begin with a fragmentation step to produce smaller RNA using RNase III or sonication [6]. Based on our results and those of Liu *et al*., fragmentation methods that tend to leave a 5’ ss extension near a ds region should be avoided because 5’ adapter ligation will be impaired and bias introduced. RNase III has been widely adopted, though it is not without bias itself [6]. Since it cuts RNA preferentially within ds regions, it is possible that some fragments are excluded from the cDNA libraries due to failed 5’ adapter ligation. However, since long ds regions are likely to be unusual in mRNA fragments, and cuts made a little farther away may not experience the 5’ adapter ligation problem, it should not lead to loss of entire exons, but could explain small gaps in tiled reads alignment. However, in the case of small RNA-seq when one does not know anything about the secondary structure of an RNA collection to be sequenced, and especially when preparing the library without monitoring each step, we recommend using a ribosome-profiling-based method, like Coligo-seq, which we show can be easily adapted to RNA without a 5’ triphosphate, to avoid systematic exclusion of structured 5’ end sequences from the cDNA library.

## Supplementary Material

Supplementary information **Figure S1**. Examples of 5’ adapter ligation optimization attempts using coligo 122 transcript as test case.
**Figure S2**. TS2126 Rnl1 (CircLigase) circularization bias experiments and sequencing fragment size.
**Figure S3**. TS2126 Rnl1 circularization bias experiment; library preparation.
**Figure S4**. TS2126 Rnl1 circularization bias analysis results.
**Figure S5**. Application of Coligo-seq to HeLa cell miRNA.
**Table S1**. Oligonucleotide sequences used in TS2126 Rnl1 bias experiments.
**Table S2**. Comparison of miRNA reads obtained using standard RNA seq method (Method1) versus Coligo-seq method (Method 2).
Supplementary information of this article can be found online at
http://www.jbmethods.org/jbm/rt/suppFiles/269.

## Figures and Tables

**Table 1.** Coligo, primer, adapter and marker sequences used for the preparation of coligo transcript libraries.

Name	Length (nt)	Sequence
Adapter1	17	5’pCTGTAGGCACCATCAAT-R-NH2
Adapter2	18	5’ACGGAATTCCTCACTrArArA
Adapter3	22	5’pCAGATCGGAAGAGCACACGTCT-R-NH2
Adapter4	21	5’AppAGATCGGAAGAGCACACGTCT-R-NH2
Adapter5	26	5’rGrUrUrCrArGrArGrUrUrCrUrArCrArGrUrCrCrGrArCrGrArUrC
RNA size marker M1	19	5’rCrGrUrArCrGrCrGrGrGrUrUrUrArArArCrGrA
RNA size marker M2	24	5’rCrGrUrArCrGrCrGrGrArArUrArGrUrUrUrArArArCrUrGrU
RTprimer1	43	5’pACGCTCTCCATGCCACAGGTrCTCCGGATTGATGGTGCCTACAG
Primer1F	21	5’CCGG3ATTGATGGTGCCTACAG
Primer1R	22	5’AGACCTGTGGCATGGAGAGCGT
LBprimer	61	5’pNNNNNNNGATCGTCGGACTGTAGAACTCTGAArCAGACGTGTGCTCTTCCGATCTNNNNNNN
RTprimer2	47	5’pGATCGTCGGACTGTAGAACTCTGAArCAGACGTGTGCTCTTCCGATCT
RTprimer3	21	5’AGACGTGTGCTCTTCCGATCT
Primer2F	50	5'AATGATACGGCGACCACCGAGATCTACACGTTCAGAGTTCTACAGTCCGA
Primer2R1 (LBprimer)	64	5'CAAGCAGAAGACGGCATACGAGATGCGGACGTGACTGGAGTTCAGACGTGTGCTCTTCCGATCT
Primer2R2 (Dcr3)	64	5'CAAGCAGAAGACGGCATACGAGATGATCTGGTGACTGGAGTTCAGACGTGTGCTCTTCCGATCT
Primer2R3	64	5'CAAGCAGAAGACGGCATACGAGATATTGGCGTGACTGGAGTTCAGACGTGTGCTCTTCCGATCT
Primer2R4	64	5'CAAGCAGAAGACGGCATACGAGATTACAAGGTGACTGGAGTTCAGACGTGTGCTCTTCCGATCT
122	89	5’pTTGCCTAGCAGTAGCTATTTAGTGTGATAATGGCGTTTGATAGTTTAGACACAAACACCATTGTCACACTCCACAGCTCTGCTAAGGAA
Dcr3	82	5’pGAAGGAAAAATCAGTTTTGCATAGATTTGCACAACTACATTCTTCTTGTAGTGCAACTATGCAAAACTGCAAACAAAAACTA

N indicates “hand-mixed” random bases. rN, ribonucleotide; R-NH2, 3’ blocking group; App, 5’ pre-adenylylated end. p indicates 5’ phosphate. Index sequences are underlined.
